# Brazos Valley Medical Association

**Published:** 1900-07

**Authors:** 


### Brazos Valley Medical Association.

The Ninth Semi-Annual Meeting of the Brazos Valley Medical Association was held at Caldwell, on the 8th and 9th of May, 1900.

About forty physicians were present, and twelve new members were received.

Cameron was selected as the next place of meeting, the second Tuesday and Wednesday in November next.

The following papers were read:

"The Baby's Second Summer." Dr. E. S. Ferguson, Cameron. "The Third Stage of Labor." Dr. W. W. Greer, Cameron. "Pernicious Vomiting of Pregnancy and the Application of Electricity in the Treatment." Dr. J. P. Oliver, Caldwell. "Summer Diarrhea in Children." Dr. J. D. Porter, Hutto. "The Chemical Laboratory as an Adjunct to Medicine." Dr. R. L. Jones, Cameron. "Scarlet Fever and Report of Cases." Dr. H. W. Cummings, Hearne. "Double Mastoid Disease Following Scarlet Fever, Operation and Recovery." Dr. Joseph Mullen, Houston. "Dysmenorrhœa." Dr. E. A. Sherrill, Hix. "Remarks Upon Treatment of Acutely Inflamed Intestines." Dr. R. E. Bledsoe, Somerville.

Election of Officers: Dr. J. P. Oliver, President, Caldwell; Dr. M. L. Langford, First Vice-President, Baileyville; Dr. E. S. Ferguson, Second Vice-President, Cameron; Dr. J. W. Hudson, Treasurer, re-election, Milano; Dr. W. B. Briggs, Secretary, re-election, Easterly.

